# Integrated analysis identifies RAC3 as an immune‐related prognostic biomarker associated with chemotherapy sensitivity in endometrial cancer

**DOI:** 10.1111/jcmm.17824

**Published:** 2023-06-29

**Authors:** Pu Huang, Yiyu Qian, Yu Xia, Siyuan Wang, Cheng Xu, Xueqiong Zhu, Qinglei Gao

**Affiliations:** ^1^ Department of Obstetrics and Gynecology the Second Affiliated Hospital of Wenzhou Medical University Wenzhou China; ^2^ National Clinical Research Center for Obstetrics and Gynecology, Cancer Biology Research Center (Key Laboratory of the Ministry of Education), Tongji Hospital, Tongji Medical College Huazhong University of Science and Technology Wuhan China; ^3^ Department of Gynecological Oncology, Tongji Hospital, Tongji Medical College Huazhong University of Science and Technology Wuhan China

**Keywords:** chemotherapy sensitivity, endometrial cancer, oncogene, RAC3, tumour immune microenvironment

## Abstract

Endometrial cancer (EC) is one of the most common gynaecological malignant tumours with a high incidence, leading to urgent demands for exploring novel carcinogenic mechanisms and developing rational therapeutic strategies. The rac family of small GTPase 3 (RAC3) functions as an oncogene in various human malignant tumours and plays an important role in tumour development. However, the critical roles of RAC3 in the progression of EC need further investigation. Based on TCGA, single‐cell RNA‐Seq, CCLE and clinical specimens, we revealed that the RAC3 was specifically distributed in EC tumour cells compared to normal tissues and functioned as an independent diagnostic marker with a high area under curve (AUC) score. Meanwhile, the RAC3 expression in EC tissues was also correlated with a poor prognosis. In detail, the high levels of RAC3 in EC tissues were reversely associated with CD8^+^T cell infiltration and orchestrated an immunosuppressive microenvironment. Furthermore, RAC3 accelerated tumour cell proliferation and inhibited its apoptosis, without impacting cell cycle stages. Importantly, silencing RAC3 improved the sensitivity of EC cells to chemotherapeutic drugs. In this paper, we revealed that RAC3 was predominantly expressed in EC and significantly correlated with the progression of EC via inducing immunosuppression and regulating tumour cell viability, providing a novel diagnostic biomarker and a promising strategy for sensitizing chemotherapy to EC.

## 1 INTRODUCTION

As the most common gynaecological tumour originating from the endometrial epithelium, endometrial cancer (EC) was diagnosed in more than 415,000 women in 2020, with the majority of cases occurring in postmenopausal women.^1^, ^2^ More worrisome is that EC‐related mortality increased by an average of 1.9% per year.^3^ However, the five‐year relative survival rate of patients with high‐grade or advanced‐stage EC has still not experienced any substantial improvement for decades.^4^

That earlier diagnosis produces a better prognosis is true for EC patients, while the recent diagnostic biomarkers depending on carbohydrate antigen 125 (CA125) and human epididymis protein 4 (HE4) have poor sensitivity and specificity for early EC detection.^5^, ^6^ Chemotherapy and hormone therapy remains the first‐line treatment options for advanced and recurrent EC, yet they can potentially induce drug resistance, which in turn poses an inevitable clinical challenge.^7^ Thus, overcoming the difficulty of early diagnosis and potentiating the sensitivity of chemotherapeutic drugs for EC have been of great urgency. Accumulating evidence suggests that oncogenes play critical roles in orchestrating tumour progression and drug resistance.^8^ However, reports on the role of novel oncogenes in EC are still scarce.

RAC3, a member of the Rho GTPases family, participates in cytoskeleton formation, cellular and developmental biology and pathological processes.^9^ Meanwhile, evidence illustrated that the RAC3 was highly expressed in a variety of human cancers, such as breast cancer,^10^ lung cancer^11^ and bladder cancer.^11^ Furthermore, RAC3 inhibited apoptosis and promoted tumour invasion, high expression of which indicated a poor prognosis for breast cancer.^12^, ^13^ However, few studies have investigated its prognostic implication and clinical significance in EC.^14^, ^15^

In the present research, we revealed that RAC3 was accumulated in EC and served as a reliable diagnostic marker with a high AUC value. Subsequently, RAC3 was associated with poor prognosis in EC via its immunosuppressive phenotype and the regulation of tumour cell viability. Finally, we figured out that RAC3 overexpression enhanced the chemo‐resistance of EC cells. In conclusion, our study highlighted RAC3 as a potential diagnostic and therapeutic target for EC patients.

## 2 METHODS AND MATERIALS

### 2.1 Data collection

RNA‐Seq and clinical information for 552 EC tissues and 35 normal tissues were downloaded from TCGA (https://gdc.cancer.gov/). The published single‐cell RNA‐Seq of EC patients were downloaded from ZENDO (https://zenodo.org/record/3937811, EGAS00001004466). RNA‐Seq data of 25 EC cell lines were obtained from CCLE (https://sites.broadinstitute.org/ccle/).

### 2.2 Tissue collection

Normal endometriums were obtained from eight women with uterine curettage. The tumour tissues were recruited from 61 patients with EC who underwent tumorectomy (Table 1). The diagnosis was confirmed by two experienced pathologists. This project was approved by the Ethics Committee of Tongji Hospital, Tongji Medical College, Huazhong University of Science and Technology.

**TABLE 1:** Characteristics of recruited patients.

Characteristic	Endometrial cancer	Normal control
*n*	61	8
Age (years, mean ± SD)	57.5 ± 9.03	54.5 ± 8.75
Histological type, *n* (%)		
Endometrioid	55 (90.1%)	–
Serous	3 (5%)	–
Mixed	1 (1.6%)	–
Undifferentiated	2 (3.3%)	–
Clinical stage, *n* (%)		
Stage IA	185 (33.5%)	–
Stage IB	24 (4.3%)	–
Stage II	55 (10%)	–
Stage III‐IV	12 (2.2%)	–
Unknown	1 (1.6%)	–
Histologic grade, *n* (%)		
G1	18 (29.5%)	–
G2	29(47.5%)	–
G3	11 (18%)	–
Unknown	3 (5%)	–
MSI‐H/dMMR, *n* (%)	8 (13.1%)	–
Oestrogen receptor positive, *n* (%)	47 (77%)	–
Progesterone receptor positive, *n* (%)	43 (70.4%)	–
Lymphatic metastasis, *n* (%)	5 (8.2%)	–

*Note*: *Microsatellite instability‐High/*deficiency of mismatch repair (MSI‐H/dMMR).

### 2.3 Single‐cell RNA‐Seq analysis

Single‐cell raw data were loaded into R software (v4.0.5) and generated by the ‘Seurat’ package (v4.1.0). Following normalizing and principal components analysis (PCA), clusters were screened by Uniform Manifold Approximation and Projection (UMAP). Marker genes were selected according to (min. pct >0.25, |logFC| > 0.25). CD45 was used as a simple marker to annotate the distribution of immune and non‐immune clusters separately. The mean expression of PTPRC, COL1A1 and EPCAM was calculated to identify the immune, stromal and tumour clusters, respectively. RAC3 was also visualized by feature plots according to mean expression.

### 2.4 Kaplan–Meier plotter analysis and ROC analysis

The prognostic value of RAC3 was analysed by a Kaplan–Meier plotter (http://kmplot.com), with the hazard ratio (HR), 95% confidence interval (CI), and log‐rank *p*‐value. The receiver operating characteristic (ROC) curves were applied to determine the diagnostic accuracy using the ‘survivalROC’ package. The AUC value is the area under the ROC curve and represents the diagnostic ability of a single factor. AUC values of more than 0.9 represented high accuracy and 0.7 ≤ AUC ≤0.9 reflected moderate accuracy.

### 2.5 Gene set enrichment analysis (GSEA)

GSEA was downloaded for the analysis of different pathways related to target genes (http://software.broadinstitue.org/gsea/). Related pathways were obtained from the molecular signatures database (MSigDB).

### 2.6 Immune cell infiltration analysis

The immune cell infiltration was assessed by TIMER 2.0 (http://timer.cistrome.org/). The correlation between RAC3 and immune cell infiltration was obtained by Spearman's correlation. A correlation coefficient over 0.25 indicates a positive correlation and a correlation coefficient less than −0.25 indicates a negative correlation.

### 2.7 Cell culture

EC cell lines (HEC‐1A and HEC‐1B) were purchased from the China Center for Type Culture Collection (CCTCC). HEC‐1A cells were cultured with Dulbecco's modified Eagle's medium (DMEM; Gibco), and HEC‐1B cells were cultured with Minimum essential medium (MEM, Gibco). The medium contains 10% foetal bovine serum (FBS, Gibco), 100 U/mL penicillin, and 100 μg/mL streptomycin (Servicebio). Cells were cultured in a thermostatic incubator at 37°C and 5% CO_2_. After resuscitation, cells were used within 10–15 passages or 3 months. When cells reached 70%–80% confluence, we harvested these cells for further experiments.

### 2.8 In vitro treatment

Lipofectamine 3000 (Invitrogen) was used to transfect the small interfering (si) RNA into HEC‐1B. Plasmids were transfected with X‐tremeGENE HP (Roche) into HEC‐1A. The effect of transfection was assessed by qPCR analysis. The sequences of siRNA and plasmid were shown in the Supplementary File. The following reagents were used in this research: cisplatin, paclitaxel and doxorubicin (MedChemExpress) were used at concentrations of 10 μM (IC25, 25% inhibiting concentration), 200 nM (IC50, 50% inhibiting concentration), and 500 nM (IC25), respectively (Figures S3 and S4). Cells were treated for 48 h and analysed by apoptosis assay or cell cycle assay.

### 2.9 RNA isolation and qPCR

Total RNA was extracted using the FastPure RNA Isolation Kit V2 (Vazyme) according to the manufacturer's instructions and then reverse‐transcribed into cDNA with HiScript SuperMix (Vazyme). The qPCR analysis was performed using ChamQ Universal SYBR qPCR Master Mix (Vazyme) on LightCycler 480 (Roche). The mRNA levels of RAC3 were analysed by the 2^−ΔΔCt^ method. The primers are provided in the Supplementary Files.

### 2.10 CCK‐8 assays

EC cells were seeded into 96‐well plates at 5000 cells/well and measured every 24 h for 3–4 days. Next, the CCK8 stock (Vazyme) was diluted 1:10 in the serum‐free medium and then added to cells at a different time and incubated for 3 h. The optical density (OD) values were measured at 450 nm.

### 2.11 Apoptosis assay

The apoptosis assay was performed using FITC Annexin V Apoptosis Detection Kit I (BD Biosciences) with the staining method. The apoptosis rate (sum of Annexin V^−^/PI^+^ and Annexin V^+^/PI^+^ cell proportion) was analysed by flow cytometry (Beckman Counter) within 1 h.

### 2.12 Cell cycle assay

The cell cycle assay was performed using PI/RNase staining buffer (BD Biosciences) according to the instruction. The distribution of the cell cycle was analysed by flow cytometry (Beckman Counter).

### 2.13 Immunochemistry staining (IHC)

Following deparaffinized in xylene and rehydrated through graded alcohol solutions, paraffin‐embedded tissue slides were processed for antigen retrieval for 15 min. After blocking, slides were incubated with primary antibodies overnight at 4°C. diaminobenzidine tetrahydrochloride was then used to detect the HRP activity of the secondary antibody. The staining intensity was categorized stratified as follows: none (−, 0), faint (+, 1, yellow staining), moderate (++, 2, light brown staining) and strong (+++, 3, dark brown staining), the staining percentage of RAC3 was stratified as follows: 0 (0%), 1 (1–10%), 2 (11–50%), 3 (51–70%) and 4 (≥ 71%).^16^ IHC score of 0–7 is the sum of the staining percentage value and staining intensity value. The positive area and cell density in each slice were quantified by ImageJ analysis (NIH, Bethesda). The following antibodies were used in this research: Rabbit anti‐human RAC3 antibody (1:400 dilution; Abcam) and rabbit anti‐human CD8 antibody (1:200 dilution; Abcam).

### 2.14 Western blot analysis

EC cells were harvested and lysed in RIPA lysis buffer (Servicebio). After extraction, the total protein was measured by the Bicinchoninic Acid (BCA) protein assay kit (Beyotime), mixed with 5 × loading buffer, and then boiled in 100°C water for about 10 min. Subsequently, the well‐prepared samples were loaded into SDS‐PAGE with constant voltage and transferred to Polyvinylidene fluoride (PVDF) membranes with a constant current. Following blocking with 5% skim milk (Servicebio) for about 1 h at room temperature, the membranes were incubated with primary antibodies overnight at 4°C. The following primary antibodies were used in this research: Rabbit anti‐human RAC3 antibody (1:1000 dilution; Abcam) and rabbit anti‐human β‐actin antibody (1:1000 dilution; Abcam). Then, membranes were incubated with the appropriate Horseradish Peroxidase (HRP)‐conjugated secondary antibodies (1:5000 dilution; Abcam) for 1 h at room temperature. Finally, protein bands were detected using the chemiluminescence detection system (Bio‐Rad).

### 2.15 Statistical analysis

All data were analysed using SPSS version 19.0 (IBM SPSS). Unpaired *t*‐tests were used to compare differences between the groups. Pearson tests were used to analyse the correlation between two paired factors. Data obtained from Kaplan–Meier plotter presented as hazard ratio (HR) and *p* values or Cox *p* values upon the log‐rank test. GSEA analysis presented as Normalized enrichment score (NES), False discovery rate, and *p* values. *p* < 0.05 is considered to be of statistical significance.

## 3 RESULTS

### 3.1 The highly expressed RAC3 in EC functions as a potential diagnostic marker and an independent prognostic marker

**FIGURE 1:**
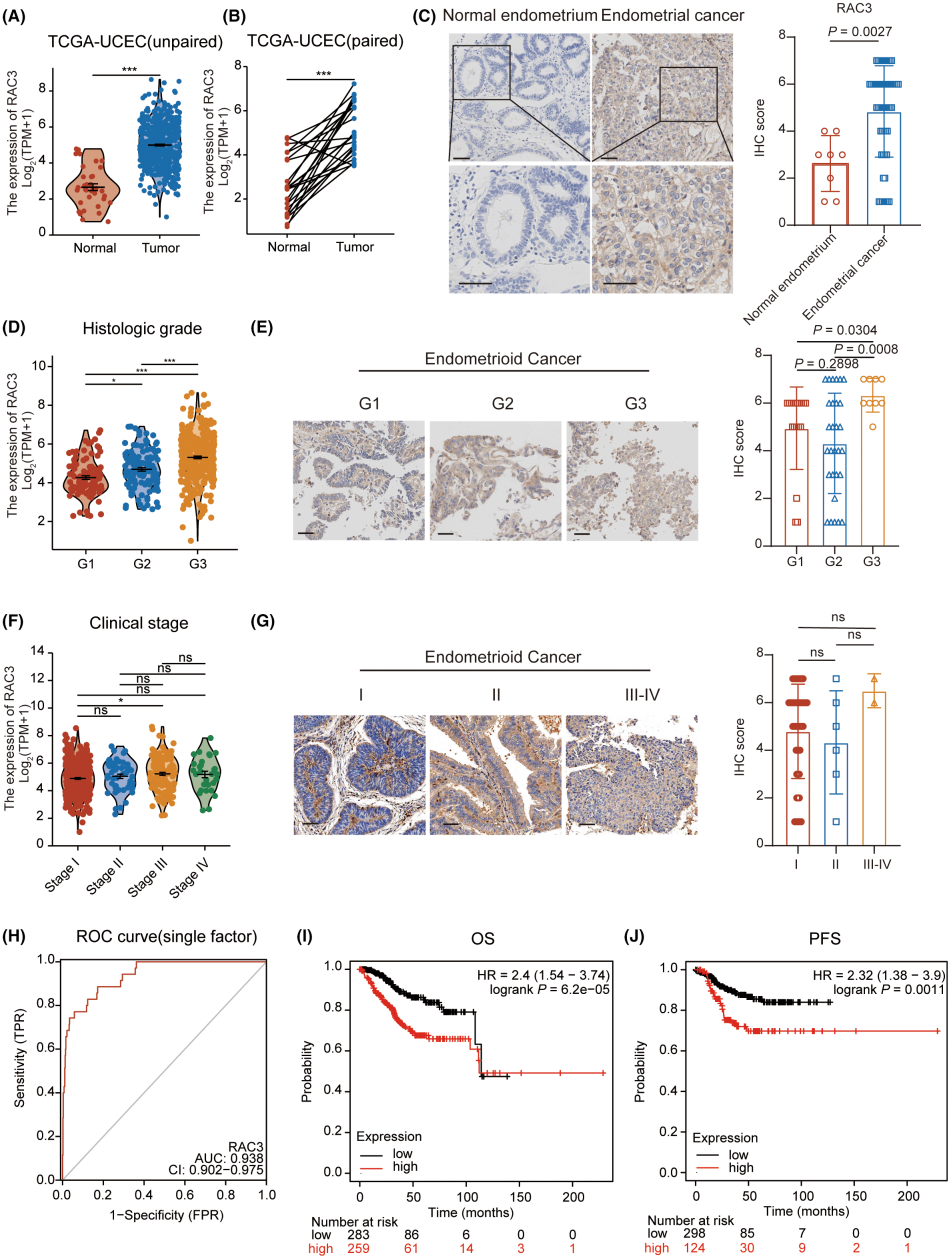
Upregulated expression of RAC3 in EC patients with clinical relevance. (A) RAC3 expression of unpaired EC and normal endometrium tissues in the TCGA cohort. (B) RAC3 expression of paired EC and peritumoral tissues in the TCGA cohort. (C) Immunohistochemical staining for RAC3 in EC and normal endometrium tissues with statistical analysis (bar, 100 μm). (D) RAC3 expression of EC patients with different histologic grades in the TCGA cohort. (E) Immunohistochemical staining for RAC3 in different histologic grades with statistical analysis (bar, 100 μm). (F) RAC3 expression of EC patients with different clinical stages in the TCGA cohort. (G) Immunohistochemical staining for RAC3 in different clinical stages with statistical analysis (bar, 100 μm). (H) Receiver operating characteristic (ROC) curves of RAC3 for predicting survival in the TCGA cohort. (I)‐(J) The correlation between RAC3 expression level and the prognosis of OS and PFS in the TCGA cohort. *p* value was denoted as *p < 0.05, **p < 0.01, ***p < 0.001, ****p < 0.0001.

RAC3 was originally identified in a screening for RAC family members involved in EC analysis (Figure S1). Among the 33 tumour subtypes archived in TCGA cohorts, 15 kinds had statistically significant RAC3 expression differences between tumour and normal tissues, including EC (Figure S1). In the TCGA‐UCEC cohort, we found that RAC3 is highly expressed in EC tissues by comparison with paired or unpaired normal endometrium (Figure 1A,B). To further validate these findings, we collected clinical specimens of EC (*n* = 61, Table 1) and normal endometrium (*n* = 8, Table 1) and evaluated the expression levels of RAC3 protein. The results indicated that RAC3 was highly expressed in tumour tissues but had limited expression in normal tissues (Figure 1C). A total of 552 TCGA patients with EC were divided into the high RAC3 expression group and the low RAC3 expression group. The RAC3 expression levels did not significantly correlate with age, BMI, clinical stage and tumour invasion (Table 2). Statistical analysis showed a significant correlation between RAC3 expression levels and clinical characteristics, including histological type, histologic grade and overall survival (OS) event. Patients with high‐grade EC had significantly higher RAC3 expression levels than patients with low‐ or moderate‐grade (Figure 1D,E). In addition, the results also showed the RAC3 expression levels were not related to the clinical stage (Figure 1F,G). Furthermore, RAC3 had good diagnostic accuracy in EC, indicated by the area under the curve (AUC) value of 0.938 (Figure 1H). Kaplan–Meier survival curve elucidated that EC patients with low‐RAC3 expression had significantly longer survival time than those with high‐RAC3 (Figure 1I,J). These data showed that RAC3 was a potential diagnostic marker and prognostic factor for EC patients.

**TABLE 2:** Characteristics of patients from TCGA cohort.

Characteristic	Low expression of RAC3	High expression of RAC3	*p*
*n*	276	276	
Age, *n* (%)			0.069
≤60	114 (20.8%)	92 (16.8%)	
>60	161 (29.3%)	182 (33.2%)	
BMI, *n* (%)			0.136
≤30	99 (19.1%)	113 (21.8%)	
>30	165 (31.8%)	142 (27.4%)	
Clinical stage, *n* (%)			0.093
Stage I	185 (33.5%)	157 (28.4%)	
Stage II	24 (4.3%)	27 (4.9%)	
Stage III	55 (10%)	75 (13.6%)	
Stage IV	12 (2.2%)	17 (3.1%)	
Histological type, *n* (%)			< 0.001
Endometrioid	234 (42.4%)	176 (31.9%)	
Mixed	7 (1.3%)	17 (3.1%)	
Serous	35 (6.3%)	83 (15%)	
Histologic grade, *n* (%)			< 0.001
G1	78 (14.4%)	20 (3.7%)	
G2	74 (13.7%)	46 (8.5%)	
G3	124 (22.9%)	199 (36.8%)	
Tumour invasion, *n* (%)			0.070
<50	142 (30%)	117 (24.7%)	
≥50	99 (20.9%)	116 (24.5%)	
OS event, *n* (%)			0.001
Alive	244 (44.2%)	214 (38.8%)	
Dead	32 (5.8%)	62 (11.2%)	

### 3.2 RAC3 is mainly distributed in tumour epithelial cells

**FIGURE 2:**
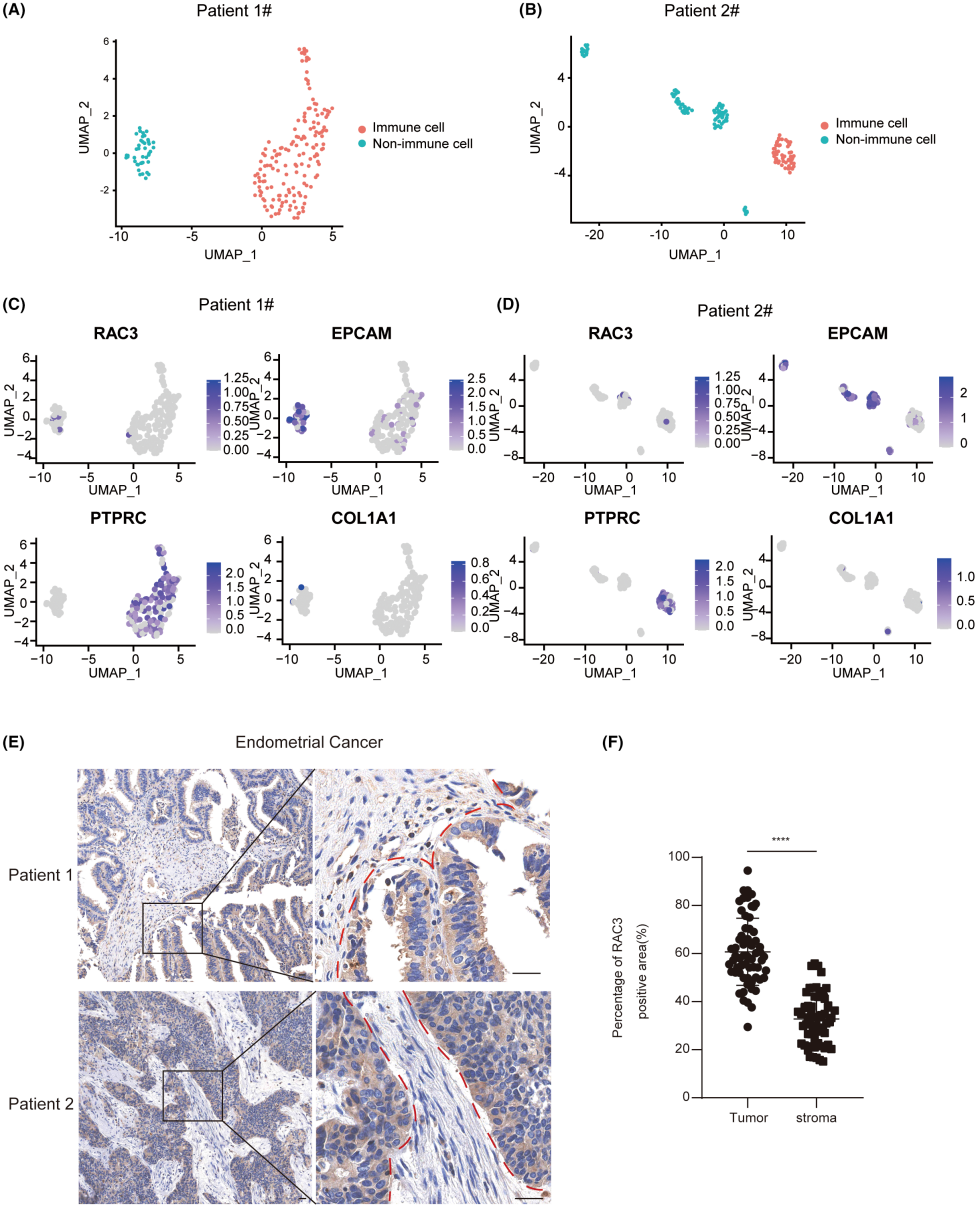
The main expression location of RAC3 in EC patients. (A, B) UMAP presentation of two distinct clusters from patients with labels and coloured according to immune cell and non‐immune cell. (C, D) Feature plot of RAC3, EPCAM, PTPRC, and COL1A1 on the UMAP projection. (E) Immunohistochemical staining for RAC3 with distinct tumour‐stromal border in recruited EC patients (bar, 100 μm). The dotted line demarcates the tumour and stroma region. (F) Percentage of RAC3 positive area between tumour and stroma area with statistical analysis. *p* value was denoted as *p < 0.05, **p < 0.01, ***p < 0.001, ****p < 0.0001.

The previous subsections covered the RAC3 expression at the tissue level. To explore the principal distribution of RAC3, we employed the single‐cell RNA‐Seq dataset of two EC patients (Patient1#–2#). We yielded two distinct cell populations on a UMAP plot including immune cells and non‐immune cells (Figure 2A,B). Additionally, we subdivided cell populations into three subpopulations. The results indicated that RAC3 was mainly co‐localized with EPCAM, a considerable marker highly expressed in epithelial tumour cells (Figure 2C,D). To analyse RAC3 location intuitively, we detected its expression in collected specimens with IHC staining. The results showed that RAC3‐positive cells were mainly enriched in tumour epithelial regions than in stromal regions. (Figure 2E,F). To sum up, the above results suggested that RAC3 was predominantly abundant in tumour epithelial cells.

### 3.3 The highly expressed RAC3 in EC is correlated with an immunosuppressive tumour microenvironment

**FIGURE 3:**
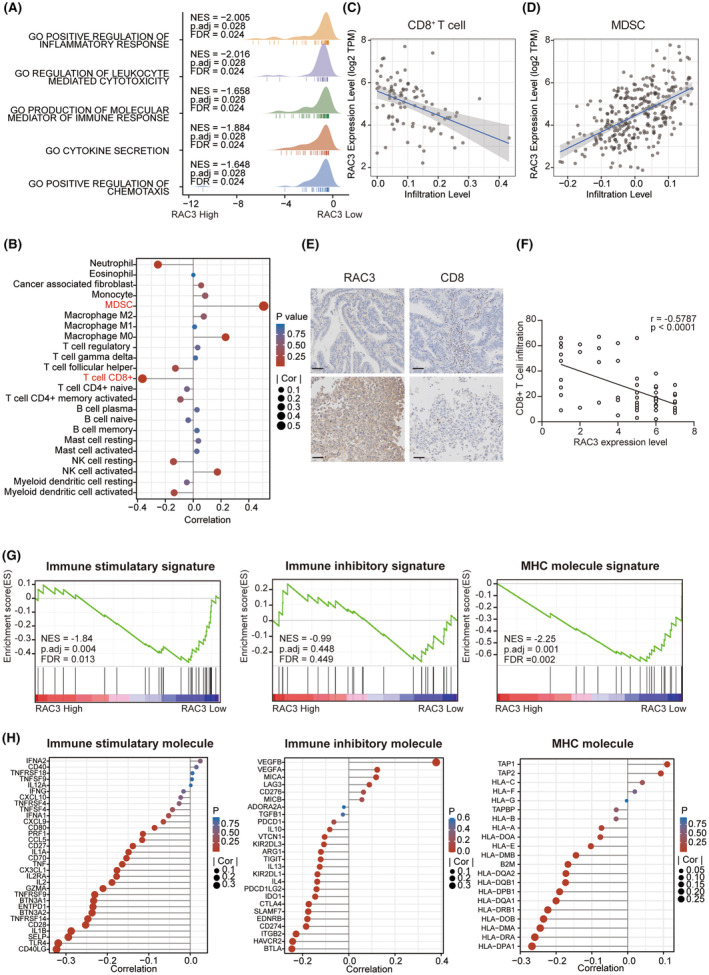
Tumour immune characteristics in distinct RAC3 expression. (A) Dissection of RAC3‐associated immune signalling pathways by GSEA Analysis of 25 EC cell lines from the CCLE database. (B) Association between RAC3 and 23 immune cell infiltration levels in EC in the TCGA cohort. (C) The correlations between RAC3 expression level and CD8^+^T cell in EC in the TCGA cohort. (D) The correlations between RAC3 expression levels and MDSC in EC in the TCGA cohort. (E) Immunohistochemical staining for CD8 with different expression levels of RAC3 in the EC patients (bar, 100 μm). (F) The correlations between RAC3 expression levels and CD8^+^ T cell infiltration level per 40× microscope in EC patients. (G) Dissection of RAC3‐associated immune‐related signatures by GSEA Analysis of 25 EC cell lines from the CCLE database. (H) The correlation between RAC3 expression level and immune‐related molecules in EC in the TCGA cohort. *p* value was denoted as *p < 0.05, **p < 0.01, ***p < 0.001, ****p < 0.0001.

Next, we focused on how RAC3 reshaped the immune‐microenvironment of EC by analysing the RNA‐Seq of 25 EC cell lines from CCLE. The results indicated that the low expression of RAC3 was correlated with immune‐related pathways, including regulation of inflammatory response, leukocyte‐mediated cytotoxicity, production of molecular mediator of the immune response, cytokine secretion and regulation of chemotaxis (Figure 3A). The relationship between RAC3 and different types of immune cells was further explored by TIMER 2.0 databases (Figure 3B–D). We found that the expression levels of RAC3 were correlated with the infiltration of Myeloid‐derived suppressor cells (MDSC) positively. Importantly, the RAC3 expression levels were reversely associated with the abundance of CD8^+^ T cells, which was verified with clinical specimens' IHC staining (Figure 3E,F). Subsequently, we employed three immune‐related signatures, including immunostimulators, immune inhibitors, and major histocompatibility antigen (MHC) molecules to explore how RAC3 modulated immune phenotypes. GSEA analysis demonstrated that RAC3 was reversely correlated with immunostimulatory and MHC molecules, and non‐significantly correlated with immune inhibitors (Figure 3G). The above results were also confirmed by a single gene correlation analysis in the TCGA cohort (Figure 3H). Thus, the high‐level expression of RAC3 in EC orchestrated an immunosuppressive microenvironment and promoted tumour progression.

### 3.4 RAC3 accelerates the proliferation and strengthens the chemo‐resistance of endometrial cancer cells

**FIGURE 4:**
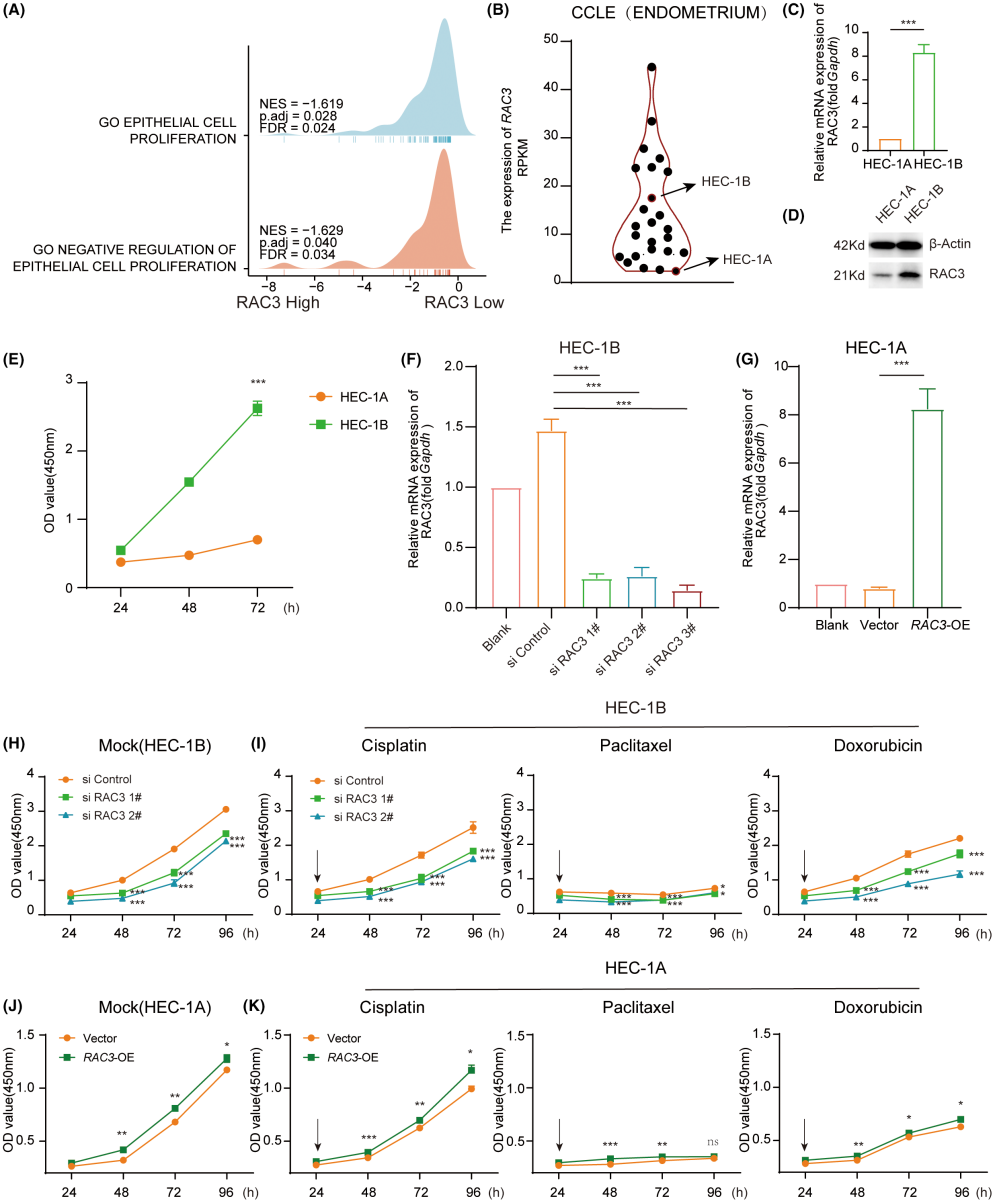
RAC3 regulated cell proliferation and attenuated drug resistance of Endometrial Cancer Cells. (A) Dissection of RAC3‐associated cell proliferation pathways by GSEA analysis of 25 EC cell lines from the CCLE database. (B) The expression level of 25 EC cell lines from the CCLE database. (C) The mRNA expression of RAC3 in HEC‐1A and HEC‐1B was determined by qPCR normalized with GAPDH mRNA. (D)The protein expression of RAC3 in HEC‐1A and HEC‐1B was determined by western blotting normalized with β‐Actin. (E) CCK8 assay was employed to detect the cell viability of HEC‐1A and HEC‐1B. (F, G) Knocking down the efficiency of siRAC3 in HEC‐1B cells and overexpression of RAC3 in HEC‐1A cells determined by qPCR normalized with GAPDH mRNA. (H, I) HEC‐1B cells were transfected with si Control, si RAC3 1# and si RAC3 2#. CCK8 assay was employed to detect cell proliferation with mock, cisplatin, paclitaxel, and doxorubicin for 24–72 h. (J, K) HEC‐1A cells were transfected with empty pCDNA3.1 vector (Vector) and pcDNA3.1‐RAC3 (RAC3‐OE). CCK8 assay was employed to detect the proliferation with mock, cisplatin, paclitaxel, and doxorubicin for 24–72 h. *p* value was denoted as *p < 0.05, **p < 0.01, ***p < 0.001, ****p < 0.0001.

To further investigate how RAC3 modulated tumour cell growth, the CCLE data involving 25 EC cell lines with different RAC3 expression levels were analysed by GSEA. The results indicated that the RAC3‐low group was related to the negative regulation of the epithelial cell proliferation gene set (Figure 4A). Moreover, a RAC3‐high cell line (HEC‐1B) and a RAC3‐low (HEC‐1A) were chosen according to CCLE and verified by Q‐PCR and western blotting (Figure 4B–D). We found that HEC‐1B harboured a higher intrinsic proliferative rate than HEC‐1A by CCK8 assay (Figure 4E). Furthermore, the transcription level of RAC3 was modified by silencing RNA or overexpression plasmid to verify the cell viability modified by RAC3 (Figure 4F,G). Consistent with the phenotypes mentioned above, silencing RAC3 constrained the growth of HEC‐1B, while overexpressing RAC3 promoted the growth of HEC‐1A (Figure 4H,J). Importantly, to further explore whether RAC3 lent itself to the chemo‐sensitivity of EC, HEC‐1B and HEC‐1A were administrated with cisplatin, paclitaxel or doxorubicin upon silencing or overexpressing RAC3. We found that silencing RAC3 in HEC‐1B promoted the proliferation inhibition of chemotherapy drugs while overexpressing RAC3 in HEC‐1A attenuated the proliferation inhibition of chemotherapy drugs in varying degrees (Figure 4I,K). Additionally, RAC3 was demonstrated to have little effect on the cell cycle of EC by PI staining (Figure S2). Together, RAC3 promoted EC cell viability and restricted the inhibited proliferation caused by chemotherapy drugs.

### 3.5 RAC3 inhibits intrinsic cell apoptosis and drug‐induced cell death

**FIGURE 5:**
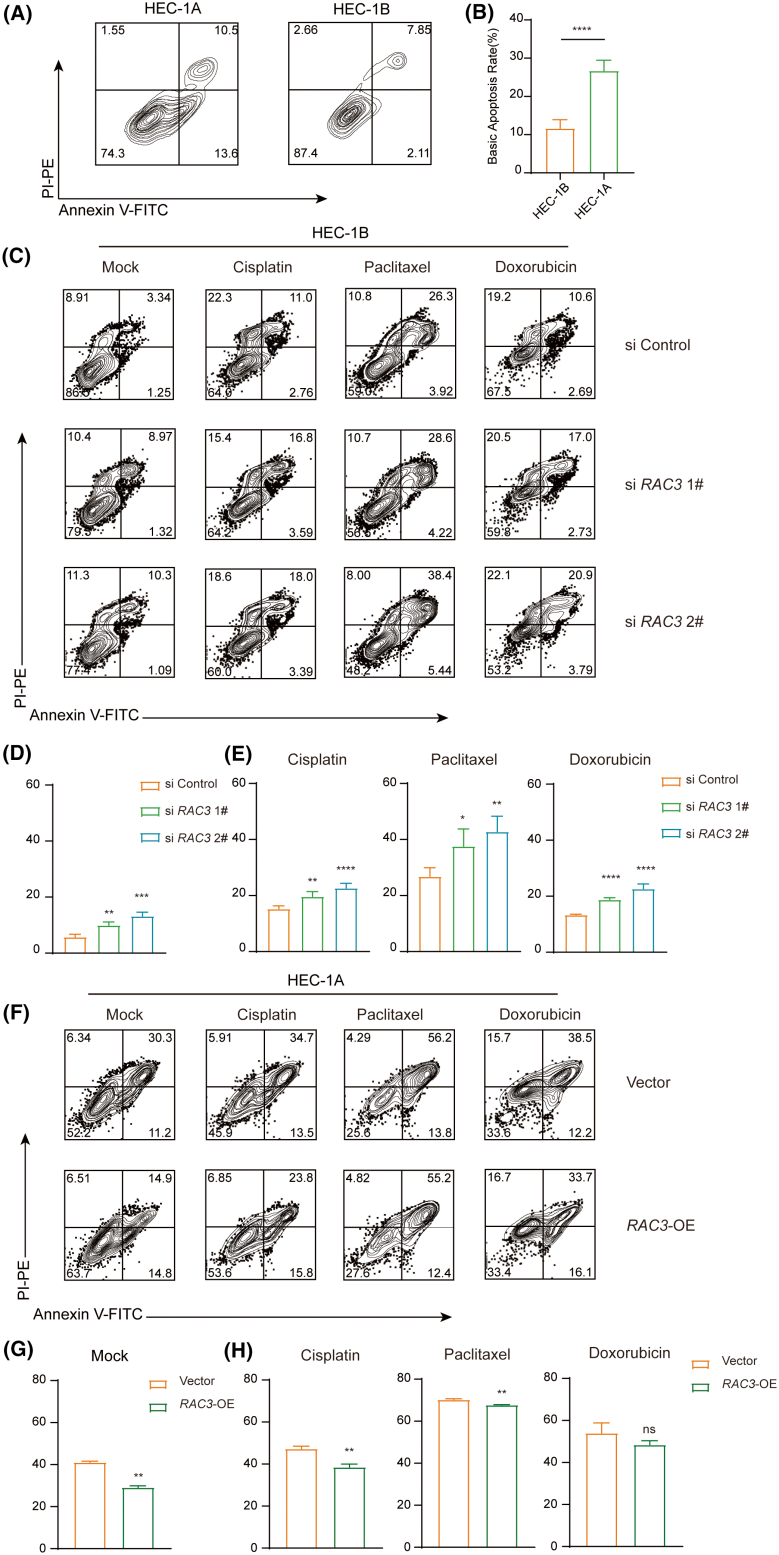
RAC3 affects cell viability by inducing apoptosis. (A, B) The basic apoptosis rate between HEC‐1A and HEC‐1B was determined by apoptosis assay. (C–E) HEC‐1B cells were transfected with si Control, si RAC3 1# and si RAC3 2#. Apoptosis assay was employed to detect apoptosis rate with mock, cisplatin, paclitaxel, and doxorubicin for 48 h. (F)‐(H) HEC‐1A cells were transfected with empty pCDNA3.1 vector (Vector) and pcDNA3.1‐RAC3 (RAC3‐OE). Apoptosis assay was employed to detect apoptosis rate with mock, cisplatin, paclitaxel and doxorubicin for 48 h. *p* value was denoted as *p < 0.05, **p < 0.01, ***p < 0.001, ****p < 0.0001.

According to apoptosis‐related GSEA analysis, RAC3 also modulated the apoptosis process of EC tumour cells (Figure S2). Next, we detected the intrinsic apoptotic rate of EC cell lines and the results indicated that HEC‐1B harboured a higher intrinsic apoptotic rate than HEC‐1A by apoptotic rate analysis (Figure 5A). To further verify the apoptosis process affected by RAC3, the transcription level of RAC3 was modified with silencing RNA or overexpression plasmid. The results showed that silencing RAC3 promoted the apoptosis of HEC‐1B while overexpressing RAC3 constrained the apoptosis of HEC‐1A (Figure 5C,D,F,G). Meanwhile, RAC3‐silenced cells displayed increased sensitivity to the above‐mentioned chemotherapy drugs in varying degrees (Figure 5C,E). Conversely, RAC3‐overexpressed cells exhibited enhanced resistance to chemotherapy drugs (Figure 5F,H). Overall, RAC3 inhibited EC cell apoptosis and restricted cell death caused by chemotherapy drugs.

## 4 DISCUSSION

In our research, we revealed that RAC3 was predominantly expressed in EC tissues by comparison with normal endometrium and harboured a high diagnostic value. Moreover, our results suggested that RAC3 was related to poor prognosis, which might be due to immunosuppressive effects and proliferation‐promoting effects on tumour cells. Importantly, we investigated the synergistic therapeutic effect of targeting RAC3 with chemotherapeutic drugs and figured out that silencing RAC3 enhanced the sensitivity of EC to cisplatin, paclitaxel and doxorubicin. Therefore, RAC3 has the potential to be a new diagnostic/prognostic marker and a therapeutic strategy for EC treatment.

Many recent studies have reported the expression patterns and biological effects of RAC3 in various tumours, such as oesophageal, breast, bladder and gastric cancer.^17^ As early as 2007, prostate carcinomas have shown higher RAC3 expression levels than their normal controls.^18^ Analysis of the RAC3 involved in bladder cancer was carried out by Cheng et al., which showed that RAC3 was up‐regulated in bladder cancer tissues and was significantly correlated with grade and stage.^19^ In general, RAC3 exhibits high expression levels in most tumours of epithelial origin. The diagnosis between benign lesions and EC tissues is primarily detected by morphological characteristics and is upheld also by additional molecular alterations.^20^ As a consequence, more diagnostic molecules are urgently needed to help distinguish tumour tissues from benign lesions in EC. In our study, the high expression of RAC3 was analysed by RNA‐Seq and IHC staining from mRNA level and protein level, respectively. Importantly, the high percentage of tumour‐cell staining and the AUC values close to 1 supported RAC3 as a good diagnostic marker in EC.

Estimating EC patients' prognosis correctly determined the optimal treatment and the appropriate follow‐up time. In 2013, TCGA has applied multi‐platform analysis to integrate data to study and classify four types of EC including POLE acculturated, microsatellite instability hypermutated (MSI‐H), copy number abnormalities‐low (CN‐L), and copy number abnormalities‐high (CN‐H).^21^ Different types of TCGA corresponded to the different prognosis and outcomes of EC patients. In addition, novel prognostic biomarkers were continually being discovered, contributing to further subdividing of low‐ and high‐risk EC patients. We found that two distinct RAC3 expression groups identified by median cut‐off had different survival outcomes and the RAC3‐high expression group had a survival disadvantage. Previous studies have also shown that high RAC3 expression was associated with poor survival in lung adenocarcinoma, bladder cancer and breast cancer et al.^12^, ^19^, ^22^ Recent studies highlighted significant roles of the RAC3 in tumour development and progression. The tumour immune microenvironment (TIME) influences multiple parameters of the tumorigenic process.^23^ Immune cells are the fundamental ingredients of TIME, which affect the occurrence of tumours by affecting the immune cell compositions.^24^ Subsequent analysis found that RAC3 correlated with high infiltration of MDSCs and low distribution of CD8^+^ T cells. CD8^+^ T cells could kill tumour cells directly and MDSCs mediated more suppression of immune responses.^25^, ^26^ MDSCs also suppressed T cell proliferation and activation and promoted tumour cell evasion from immune surveillance.^27^, ^28^ Thus, we provided evidence for the immunosuppressive role of RAC3 in EC, and patients with high‐expressed RAC3 might not suitable for immune checkpoint blockade. From another perspective, we revealed that RAC3 regulated EC cell viability and participated in the progression of EC by promoting proliferation and inhibiting apoptosis. Overall, RAC3 was significantly correlated with the progression of EC by inducing immunosuppression and regulating tumour cell viability.

Chemotherapy and hormonal therapy occupied a vital position in the treatment of patients with advanced and recurrent EC. Once drug resistance emerges during treatment, initially responsive tumours become refractory to treatment.^7^ Therefore, drug sensitivity/resistance plays an indispensable part in the individualization of EC therapy. A previous study has reported that RAC3 affected the sensitivity to apoptosis and autophagy of colon tumour cells induced by chemotherapeutic drugs.^29^ In this study, we estimated that RAC3 increased sensitivity to classical chemotherapeutic drugs of EC cells, including cisplatin, paclitaxel and doxorubicin. From this perspective, combination therapy with chemotherapeutic drugs and RAC3‐targeted inhibitors might provide a synergistic effect for EC treatment. EHop‐016, the RAC1 and RAC3 dual‐target inhibitor, has the potential as an anti‐cancer compound to block cancer progression via multiple Rac‐directed mechanisms.^30^, ^31^, ^32^ A previous study has also shown that a combination therapy comprising EHop‐016 and cetuximab confers strong and synergistic efficacy against head and neck squamous cell carcinomas (HNSCCs) in a population‐level patient‐derived xenograft (PDX) clinical trial.^33^ Therefore, EHop‐016 may be served as a rationale for our drug candidate and combination therapy selection of EC treatment. However, there was no specific RAC3 inhibitors are currently available for clinical studies. Consequently, developing drugs that directly target RAC3 needs urgent attention for EC patients.

There were several limitations in this study. First, there was no external data to predict the prognosis of RAC3 in EC patients. Second, more experiments were needed to explain the molecular mechanisms and pathways of RAC3 in EC.

In conclusion, this study systematically revealed the expression patterns of RAC3 in EC. Our work also provided a practical biomarker for predicting the prognosis of EC and suggested that targeting RAC3 might provide new strategies for EC treatment.

## AUTHOR CONTRIBUTIONS

**Pu Huang:** Conceptualization (equal); data curation (equal); methodology (equal); project administration (equal); resources (equal); software (equal); validation (equal); visualization (equal); writing – original draft (equal). **Yiyu Qian:** Investigation (equal); methodology (equal); project administration (equal). **Yu Xia:** Conceptualization (equal); formal analysis (equal); funding acquisition (equal); supervision (equal); writing – review and editing (equal). **Siyuan Wang:** Investigation (equal); methodology (equal); project administration (equal); writing – review and editing (equal). **Cheng Xu:** Investigation (equal); methodology (equal); project administration (equal). **xueqiong zhu:** Project administration (equal); supervision (equal); writing – review and editing (equal). **Qinglei Gao:** Conceptualization (equal); funding acquisition (equal); project administration (equal); supervision (equal); writing – review and editing (equal).

## CONFLICT OF INTEREST STATEMENT

The authors have no conflict of interest to declare.

## Supporting information


Appendix S1.

Appendix S2.

## Data Availability

Publicly available datasets can be accessed by the websites provided in the methods and the data supporting this study are available from the corresponding author upon reasonable request.
