# Supplementary material for: Integrated analysis identifies RAC3 as an immune‐related prognostic biomarker associated with chemotherapy sensitivity in endometrial cancer
Source: J Cell Mol Med. 2023 Jun 29;27(16):2385–97. doi: 10.1111/jcmm.17824 (PMC10424291; doi:10.1111/jcmm.17824)
Supplement: Supplementary file 2 — Appendix S2. [file JCMM-27-2385-s001.docx]

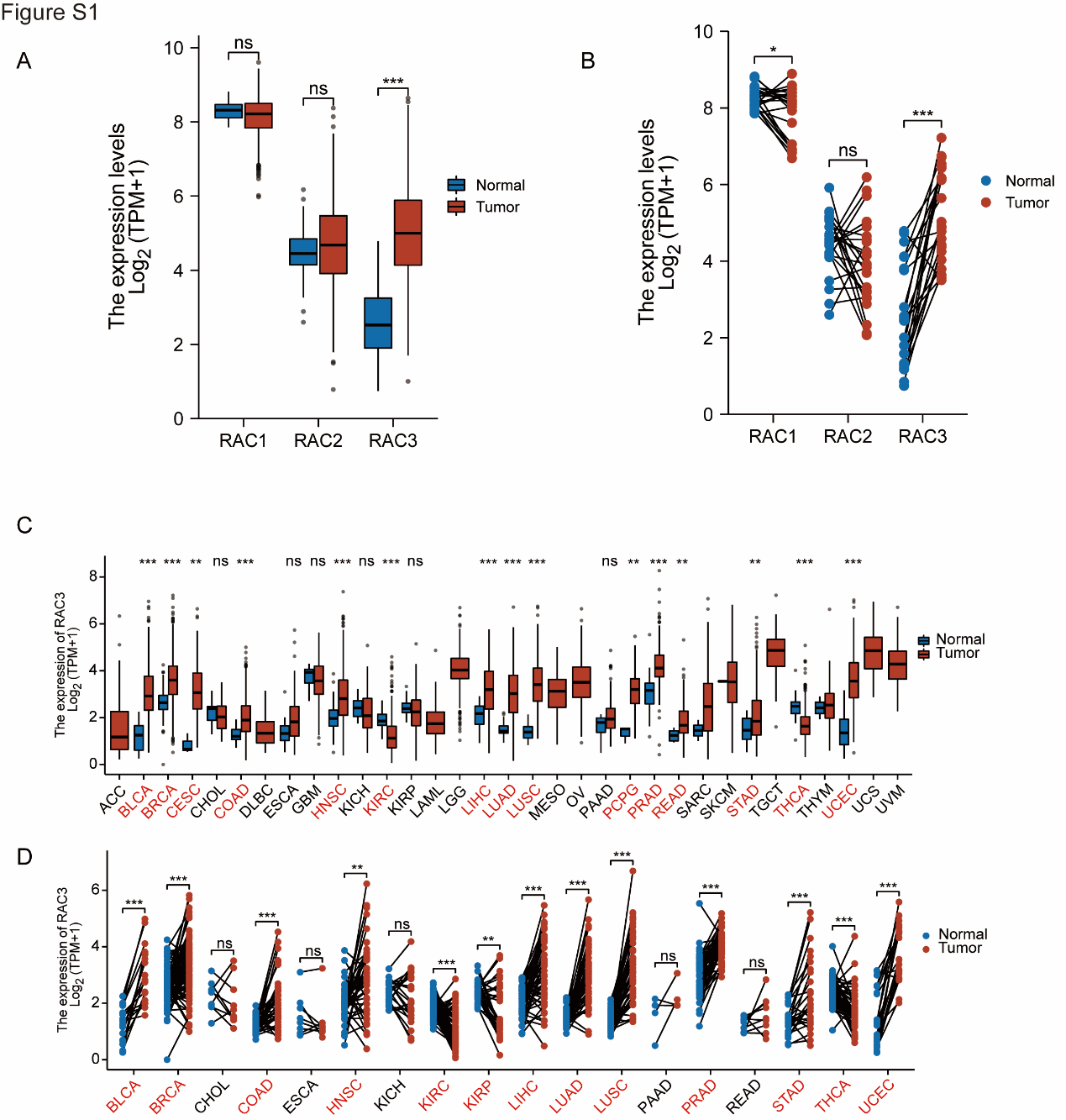


**Supplementary file 2: Figure S1.**

(A) RAC family expression characteristics of various cancers in the unpaired TCGA-UCEC cohort (RAC1, RAC2, and RAC3).

(B) RAC family expression characteristics of various cancers in the unpaired TCGA-UCEC cohort (RAC1, RAC2, and RAC3).

(C) RAC3 expression characteristics of various cancers in the unpaired TCGA cohort.

(D) RAC3 expression characteristics of various cancers in the paired TCGA cohort.

*P* value was denoted as **P*<0.05, ***P*<0.01, ****P*<0.001, *****P*<0.0001.


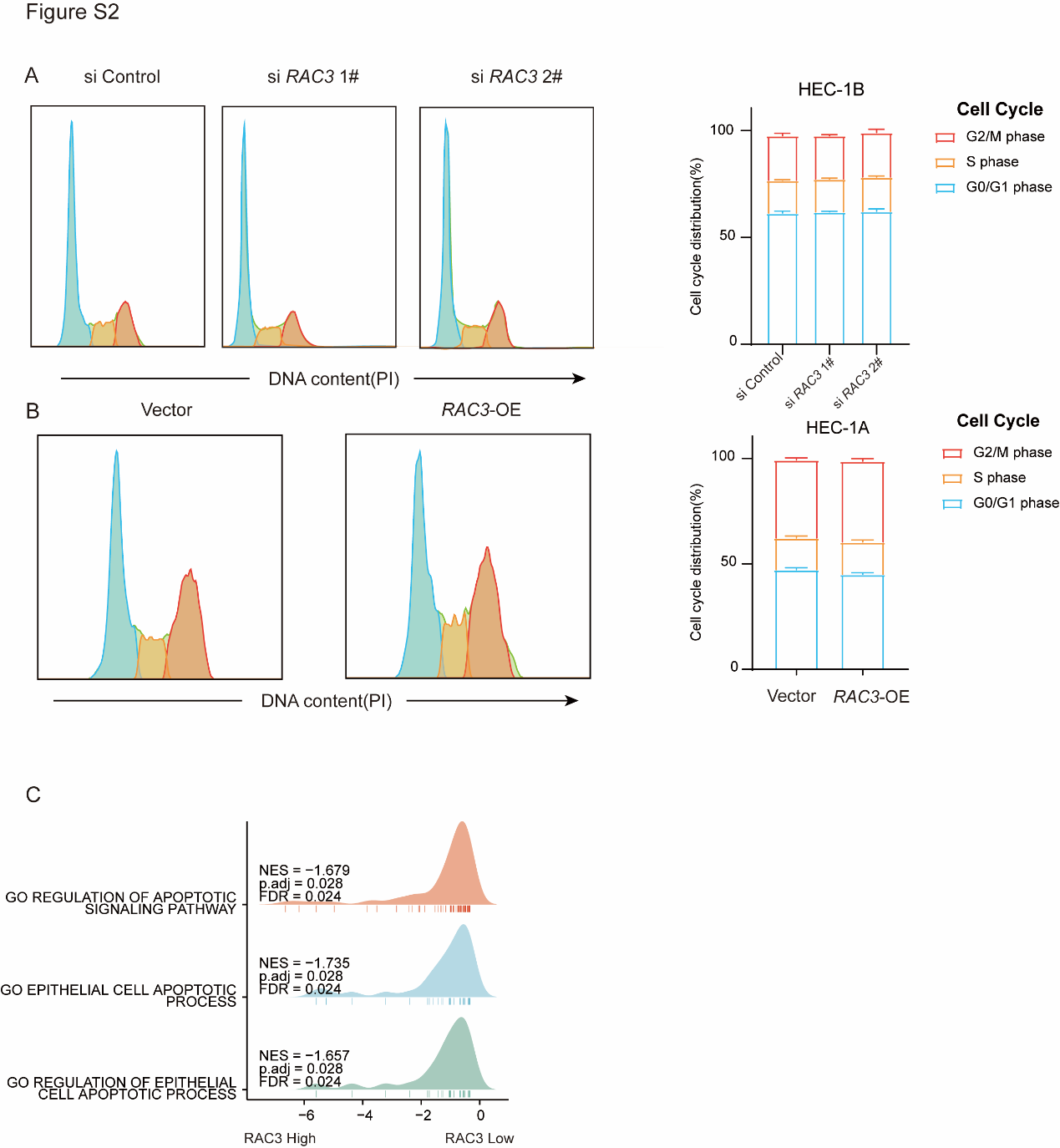


**Supplementary file 2: Figure S2.**

(A) HEC-1B cells were transfected with si Control, si RAC3 1#, and si RAC3 2#. Flow cytometry was employed to detect the cell cycle distribution.

(B) HEC-1A cells were transfected with empty pCDNA3.1 vector (Vector) and pcDNA3.1-RAC3 (RAC3-OE). Flow cytometry was employed to detect the cell cycle distribution.

(C) Dissection of RAC3-associated cell apoptosis pathways by GSEA Analysis of 25 EC cell lines from the CCLE database.

*P* value was denoted as **P*<0.05, ***P*<0.01, ****P*<0.001, *****P*<0.0001.


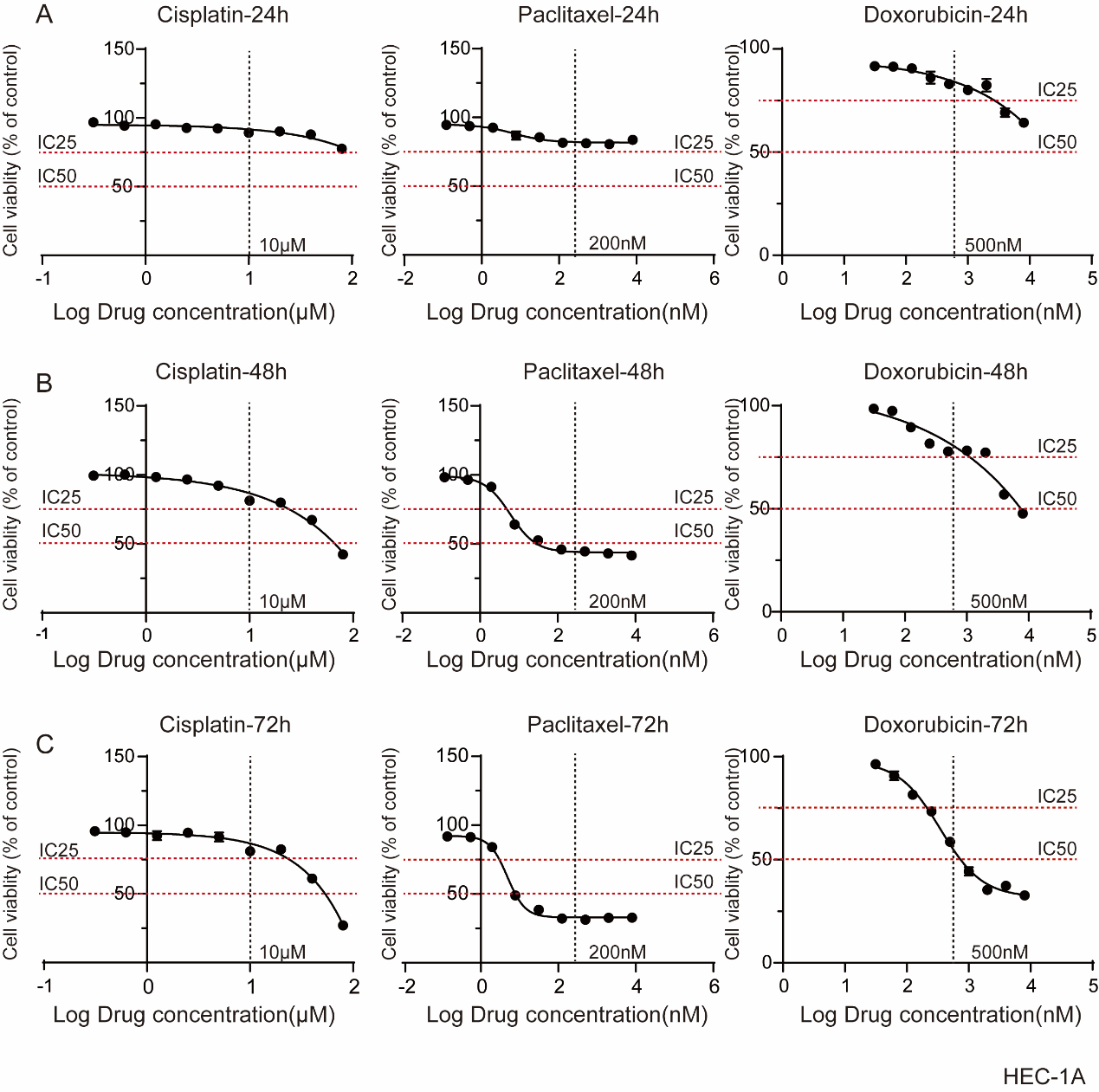


**Supplementary file 2: Figure S3.**

(A)CCK8 assay was employed to detect cell viability of HEC-1A with cisplatin, paclitaxel, and doxorubicin for 24h.

(B) CCK8 assay was employed to detect cell viability of HEC-1A with cisplatin, paclitaxel, and doxorubicin for 48h.

(C) CCK8 assay was employed to detect cell viability of HEC-1A with cisplatin, paclitaxel, and doxorubicin for 72h.

Treatment drug concentrations were as follows: cisplatin at doses of 0 , 0.3125, 0.625, 1.25, 2.5, 5, 10, 20, 40, 80 µM, paclitaxel at doses of 0, 0.122, 0.488, 1.95, 7.8125, 31.25, 125, 500, 2000, 8000 nM, doxorubicin at doses of 0, 31.25, 62.5, 125, 250, 500, 1000, 2000, 4000, 8000 nM. Red horizontal dotted line: IC25 (25% inhibiting concentration) or IC50 (50% inhibiting concentration); Black vertical dotted line: the cell viability of selected concentration.


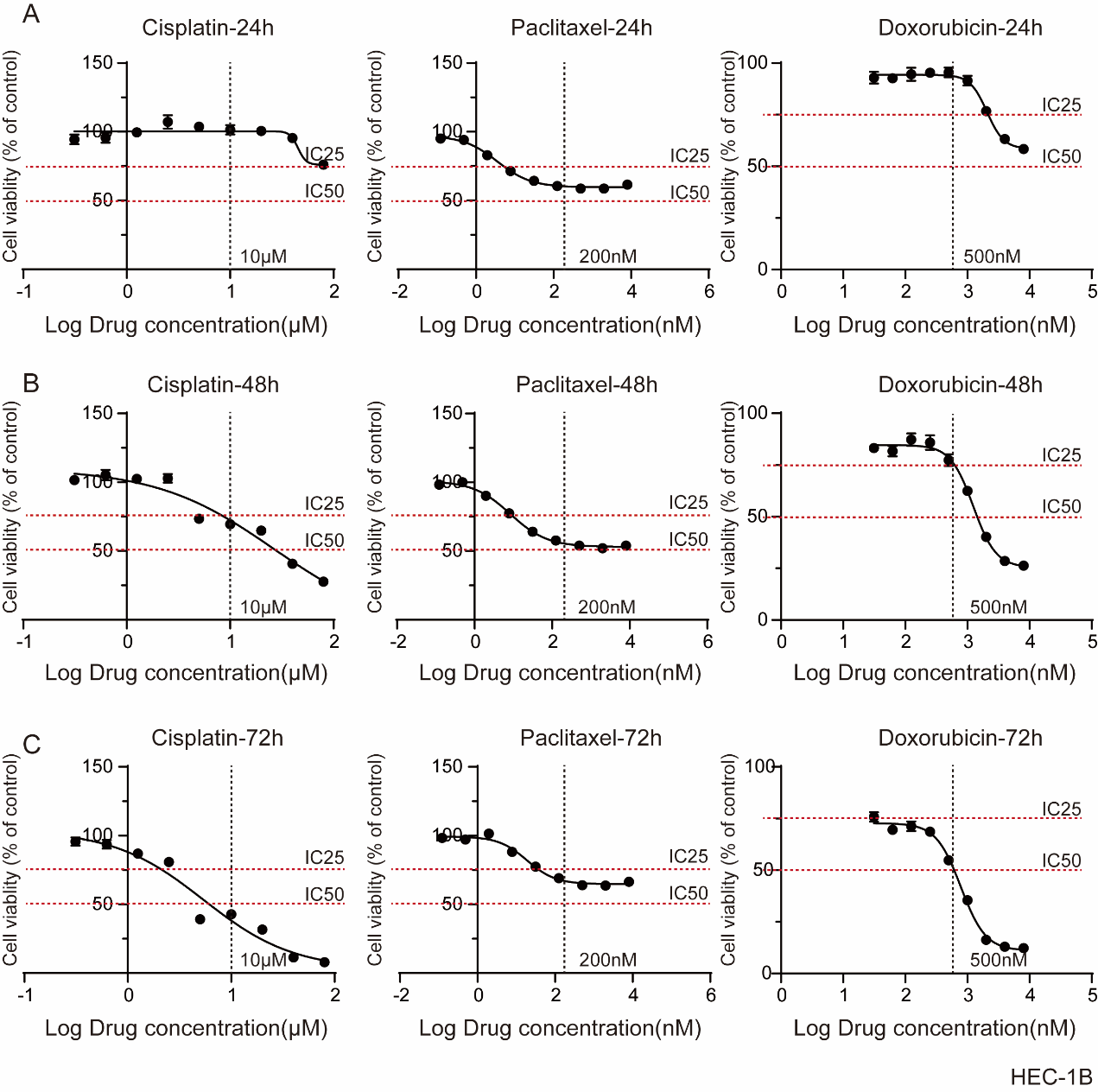


**Supplementary file 2: Figure S4.**

(A)CCK8 assay was employed to detect cell viability of HEC-1B with cisplatin, paclitaxel, and doxorubicin for 24h.

(B) CCK8 assay was employed to detect cell viability of HEC-1B with cisplatin, paclitaxel, and doxorubicin for 48h.

(C) CCK8 assay was employed to detect cell viability of HEC-1B with cisplatin, paclitaxel, and doxorubicin for 72h.

Treatment drug concentrations were as follows: cisplatin at doses of 0 , 0.3125, 0.625, 1.25, 2.5, 5, 10, 20, 40, 80 µM, paclitaxel at doses of 0, 0.122, 0.488, 1.95, 7.8125, 31.25, 125, 500, 2000, 8000 nM, doxorubicin at doses of 0, 31.25, 62.5, 125, 250, 500, 1000, 2000, 4000, 8000 nM. Red horizontal dotted line: IC25 (25% inhibiting concentration) or IC50 (50% inhibiting concentration); Black vertical dotted line: the cell viability of selected concentration.
